# Arthroscopic release of the pectoralis minor tendon from the coracoid for pectoralis minor syndrome

**DOI:** 10.1186/s40634-022-00491-x

**Published:** 2022-06-17

**Authors:** David Haeni, Natalia Martinez-Catalan, Ronda N. Esper, Eric R. Wagner, Bassem T. El Hassan, Joaquin Sanchez-Sotelo

**Affiliations:** 1 ALTIUS Swiss Sportmed Center, Rheinfelden, Switzerland; 2 Department of Orthopedic Surgery, Mayo Clinic, 200 First Street SW, Rochester, MN 55902 USA; 3 Department of Orthopedic Surgery, Hospital Fundación Jiménez Diaz, Madrid, Spain; 4 Departments of Orthopedic Surgery, Emory University, Atlanta, GA USA

**Keywords:** Pectoralis minor, Pectoralis minor syndrome, Scapular dyskinesis, Scapulothoracic abnormal motion, STAM

## Abstract

**Purpose:**

The term “*pectoralis minor syndrome*” refers to this constellation of symptoms that can occur when the pectoralis minor (Pm) is shortened and contracted. Release of the tendon of the Pm from the coracoid has been reported to provide substantial clinical improvement to patients presenting with pectoralis minor syndrome. The purpose of this study was (1) to describe the technique for endoscopic release of pectoralis minor tendon at the subdeltoid space, (2) to classify the pectoralis minor syndrome according to its severity and (3) and to report the short-term outcomes of this procedure in a consecutive series of patients diagnosed with pectoralis minor syndrome.

**Methods:**

Endoscopic release of the pectoralis minor tendon was performed in a series of 10 patients presenting with pectoralis minor syndrome. There were six females and four males with a median age at the time of surgery of 42 (range from 20 to 58) years. Four shoulders were categorized as grade I (scapular dyskinesis), and six as grade II (intermittent brachial plexopathy). Shoulders were evaluated for pain, motion, satisfaction, subjective shoulder value (SSV), quick-DASH, ASES score, and complications. The mean follow-up time was 19 (range, 6 to 49) months.

**Results:**

Arthroscopic release of the tendon of the Pm led to substantial resolution of pectoralis minor syndrome symptoms in all but one shoulder, which was considered a failure. Preoperatively, the median VAS for pain was 8.5 (range, 7–10) and the mean SSV was 20% (range, 10% - 50%). At most recent follow-up the mean VAS for pain was 1 (range, 0–6) and the mean SSV 80% (range, 50% - 90%). Before surgery, mean ASES and quick-DASH scores were 19.1 (range, 10–41.6) and 83.1 (range, 71 and 95.5) points respectively. At most recent follow-up, mean ASES and quick-DASH scores were 80.1 (range, 40–100) and 19.3 (range, 2.3–68) points respectively. No surgical complications occurred in any of the shoulder included in this study.

**Conclusions:**

Endoscopic release of the tendon of the pectoralis minor from the coracoid improves pain, function and patient reported outcomes in the majority of patients presenting with the diagnosis of isolated pectoralis minor syndrome.

**Supplementary Information:**

The online version contains supplementary material available at 10.1186/s40634-022-00491-x.

## What is known about the subject

Abnormalities of the pectoralis minor have been associated to symptoms related to scapulothoracic abnormal motion (STAM), symptomatic compression of the brachial plexus and/or axillary vessels or both. Release of the tendon of the Pm from the coracoid has been reported to provide substantial clinical improvement to patients presenting with pectoralis minor syndrome.

## What this study adds to existing knowledge

To the best of our knowledge the outcome of arthroscopic Pm tendon release has not been reported previously. We classify the pectoralis minor syndrome in three grades: Grade I (muscular Pm syndrome), Pm abnormalities associated to isolated STAM without neurologic symptoms; Grade II (neurological Pm syndrome), Pm abnormalities lead to intermittent or constant compressive brachial plexopathy, with or without STAM or vascular compression; Grade III (multifocal thoracic outlet syndrome), Pm abnormalities contribute to compression of the brachial plexus and/or brachial vessels in the setting of double or triple crush at the interscalene triangle or costoclavicular space.

## Introduction

Shortening and dysfunction of the pectoralis minor (Pm) has the potential to limit scapulothoracic motion [14], contribute to scapular dyskinesis, and lead to symptomatic compression of the brachial plexus or the axillary vessels [10]. Contracture of the Pm can be constitutional (anatomic variations) or occur secondary to a major traumatic event, microtrauma, chronic abnormal position of the scapula, or hypertrophy of the Pm in athletes [1, 2, 5, 13, 14, 19]. Interestingly, Pm abnormalities have been associated with the practice of several sports, including baseball, tennis, volleyball and weight lifting [4, 6, 11, 14].

The term “*pectoralis minor syndrome*” refers to this constellation of symptoms that can occur when the Pm is shortened and contracted. It was first described in 1967 [10] and it is a commonly used term in vascular surgery literature in the context of thoracic outlet syndrome [21]. In these circumstances, open release of the Pm has been reported as an isolated procedure, or combined with resection of the first rib or scalene muscle release [2]. In those cases in which Pm contracture has leaded to abnormal scapulothoracic motion, anterior scapular tilt with secondary impingement, and loss of motion [14], symptoms attributed to Pm contracture may respond to shoulder horizontal abduction stretching exercises [22]. For patients with persistent scapular symptoms secondary to Pm contracture despite conservative treatment, open surgical release has been reported [14].

Advances in shoulder surgery have made it appealing to consider performing release of the Pm arthroscopically. Hendrix et al. [5] described a technique to release the Pm from the articular glenohumeral space through the interval region. Over the last few years, techniques have been developed to perform extraarticular endoscopic procedures, such as arthroscopic transfer of the coracoid process (arthroscopic Latarjet procedure) [9] [12], endoscopic brachial plexus neurolysis [7], and surgical management of other less common disorders, such as subcoracoid synovial chondromatosis [3]. As such, there is increasing familiarity with endoscopic techniques to release the Pm tendon from the subdeltoid space, a step required for arthroscopic Latarjet.

We hypothesize that release of the tendon of the Pm from the coracoid provides substantial clinical improvement to patients presenting with pectoralis minor syndrome. The purpose of this study was (1) to describe our technique for endoscopic release of the tendon of the pectoralis minor at the subdeltoid space, (2) to classify the pectoralis minor syndrome according to its severity and (3) and to report short-term outcome of this procedure in a consecutive series of patients diagnosed with isolated pectoralis minor syndrome.

## Methods

### Patients

Between April 2006 and June 2019, endoscopic release of the pectoralis minor tendon was performed in a series of 11 shoulders with pectoralis minor syndrome using the same surgical technique. All patients had previously undergone conservative treatment with a specific physiotherapy program. Surgeries were performed by four different surgeons. Patients who underwent endoscopic release of the Pm tendon in conjunction with other procedures, such as shoulder reconstruction for dysplasia or formal brachial plexus neurolysis, were excluded from this study in order to isolate the potential benefit of Pm release for this particular syndrome. One patient (1 shoulder) was lost to follow-up. The remaining 10 shoulders (10 patients) are reported in this study. The median follow-up time was 14 months (6 to 49) months.

There were six females and four males with a median age at the time of surgery of 42 (range from 20 to 58) years. Three patients recalled a traumatic event (one patient had sustained a proximal humerus fracture and two patients had suffered a traction injury to their arm) possibly linked to the initiation of their symptoms. Three shoulders had undergone surgery previously for capsular release, biceps tenodesis associated to rotator cuff repair, and acromioplasty with biceps tenotomy respectively. Two patients had a concomitant capsular release together with the endoscopic pectoralis minor release, one had a suprascapular (SSN) nerve release, and one had a revision of the previously done biceps tenodesis.

**Fig. 1:**
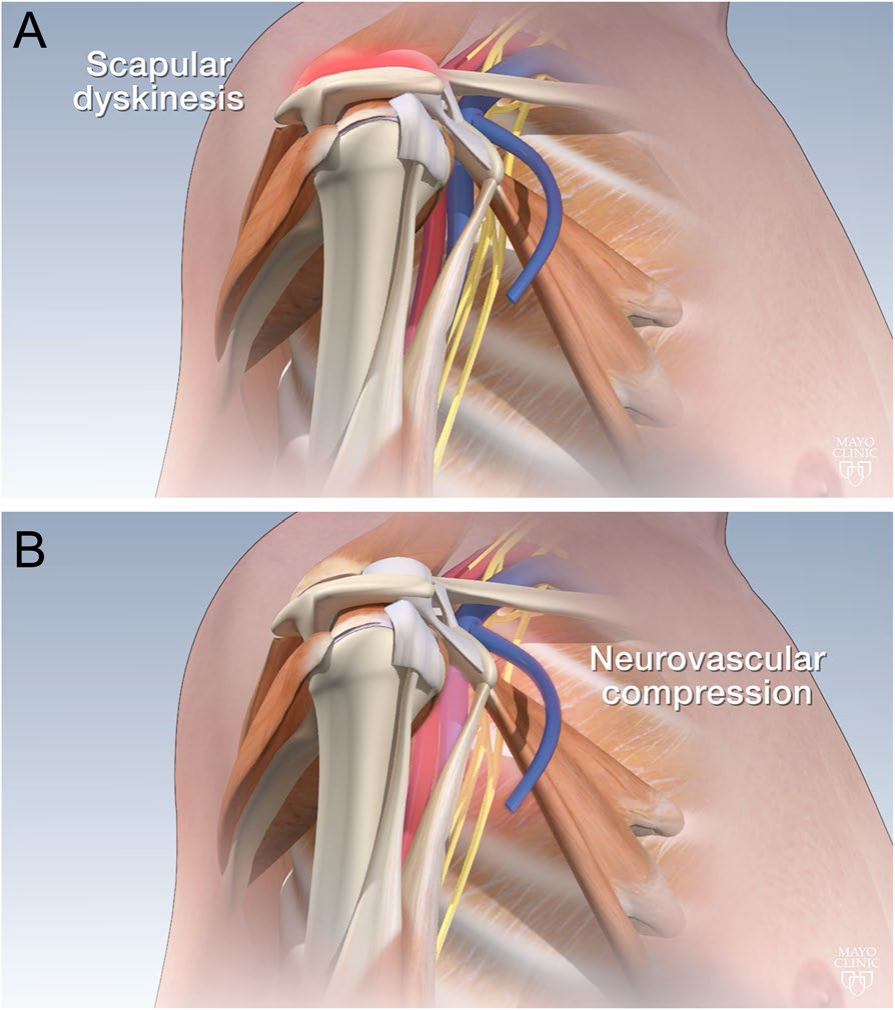
Pathologic contracture of the pectoralis minor muscle may lead to scapular dyskinesis (**A**) and/or neurovascular compression (**B**)

At the time of presentation to their treating orthopedic surgeon, all patients complained of vague pain on the anterior shoulder region centered over the coracoid process and medial to it. We classify the pectoralis minor syndrome in three grades: Grade I (muscular Pm syndrome), Pm abnormalities associated to isolated scapulothoracic abnormal motion (STAM) without neurologic symptoms; Grade II (neurological Pm syndrome), Pm abnormalities lead to intermittent or constant compressive brachial plexopathy, with or without STAM or vascular compression; Grade III (multifocal thoracic outlet syndrome), Pm abnormalities contribute to compression of the brachial plexus and/or brachial vessels in the setting of double or triple crush at the interscalene triangle or costoclavicular space. Four shoulders were categorized as grade I (Fig. 1A), and six as grade II (Fig. 1B).

Patients with grade II complained of intermittent paresthesia involving various areas of the ipsilateral upper extremity. Symptoms were reproduced with direct manual compression over the location of the pectoralis minor, with a positive Tinel sign on this location. Physical examination was completed to exclude compression of the brachial plexus at the interscalene triangle. None of the patients included in this study had a frank abnormal distal vascular examination with thoracic outlet syndrome maneuvers.

All shoulders were evaluated with plain radiographs, and all patients presented with a prior shoulder MRI. No additional studies were performed. To be considered for surgery, patients with the clinical diagnosis of pectoralis minor syndrome had to have failed 6 months of physical therapy, including shoulder horizontal abduction stretching exercises -such as the unilateral corner stretch exercise-, and scapular stabilizing exercises.

### Surgical technique

All surgeries were performed in the beach-chair position with the patient under a preoperative interscalene brachial plexus block and general anesthesia. Standard arthroscopic equipment was used, including a 30-degree arthroscope, an arthroscopic shaver, and an arthroscopic radiofrequency ablation device.

**Fig. 2:**
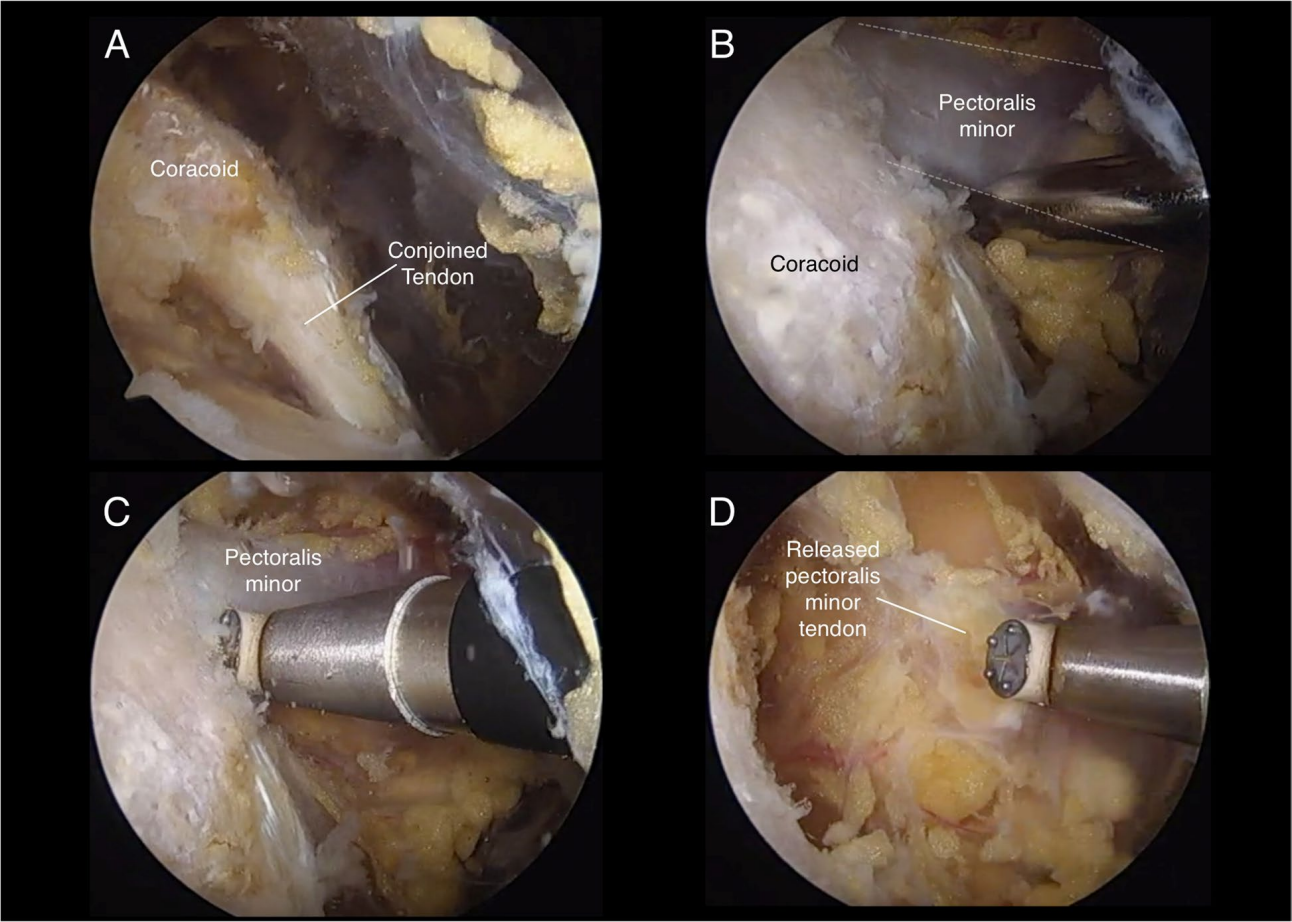
Endoscopic release of the pectoralis minor. **A** Identification of the coracoid tip and conjoined tendon from the subdeltoid space. **B** Identification and dissection of the pectoralis minor tendon. **C** Release is performed from distal to proximal with the radiofrequency ablation device oriented directly towards the coracoid process. **D** Complete release of the pectoralis minor tendon is confirmed by visualizing medial retraction of the muscle-tendon unit

After a standard diagnostic arthroscopy, bursoscopy, treatment of other associated pathology was performed when indicated. The arthroscope is placed in the lateral subacromial portal. The anterior bursal tissues are removed with a shaver or radiofrequency ablation device in the space between the anterior deltoid and the subscapularis in order to visualize the lateral aspect of the coracoid and the lateral conjoined tendon (Fig. 2A). Maintaining the shoulder in flexion with the help of an arm holder will relax the anterior deltoid and provide a larger anterior working space.

An anterior portal is then established under direct visualization at the level of the coracoid process from medial to lateral and slightly inferior to the tip of the coracoid from superior to inferior. The arthroscope is inserted through this portal, visualizing the coracoid process directly in front, the acromioclavicular ligament laterally, and the tendon of the pectoralis minor medially (Fig. 2B). A more medial working portal is established under direct vision.

A blunt instrument, such as a blunt Steinmann pin or the blunt end of a Howard periosteal elevator, is introduced through the medial working portal to identify and dissect the superior and inferior margins of the Pm tendon. Care is taken to avoid injury to the brachial plexus and vessels and to identify any abnormal adhesions. Next, a radiofrequency ablation device is used to detach the tendon of the Pm subperiosteally off the medial aspect of the coracoid with the thermal end facing superiorly and laterally (Fig. 2C-D). Complete release of the Pm tendon with medial retraction of the free Pm is confirmed under direct vision.

### Postoperative management

After arthroscopic release of the Pm tendon, patients are provided with a sling that may be used for comfort over the first day or two. Sutures are removed at week two postoperatively, and physical therapy exercises are initiated for motion and strength. Particular attention is paid to improve the strength and coordination of the periscapular muscles.

### Evaluation

All patients were evaluated prior to surgery and at most recent follow-up regarding pain level using a visual analogue scale (VAS), subjective shoulder value (SSV), shoulder ASES scores, and quick-DASH scores. A retrospective review of the chart was completed to identify any complications or reoperations. Postoperative persistence of symptoms was considered a failure.

### Statistical analysis

Data are reported using standard summary statistics, including medians for continuous variables, and counts and percentages for categorical variables. Comparisons between preoperative and postoperative values were performed using Wilcoxon test. Alpha risk was set to 5% (α = 0.05). Statistical analysis was performed with EasyMedStat (version 3.16; www.easymedstat.com).

## Results

Arthroscopic release of the tendon of the Pm led to substantial resolution of pectoralis minor syndrome symptoms in all but one shoulder, which was considered a failure. Preoperatively, the median VAS for pain was 8.5 (range, 7–10) and the median SSV was 20% (range, 10% - 50%). At most recent follow-up, the median VAS for pain was 1 with a significant improvement of − 7.5 (CIΔ 95% -8.157; − 5.643, *p* = 0.0057) and the median SSV was 80% with a significant improvement of 60% (CIΔ 95%0.452–0.688, *p* = 0.00586).

Before surgery, median ASES and quick-DASH scores were 19.1 (range, 10–42) and 83.1 (range, 71 and 96) points respectively. At most recent follow-up, there was a significant improvement of 61 points in ASES score (CIΔ 95% 41.952; 65.608, *p* <  0.01) and − 63.8 points in quick-DASH score (CIΔ 95% -71.662; − 48.318, *p* <  0.01), being median final ASES and quick-DASH scores 80.1 and 19.3 points respectively (Tables 1 and 2). No surgical complications occurred in any of the shoulder included in this study.

**Table 1:** Clinical evaluation of shoulders included in this study

Shoulder	Gender	Age (years)	FU (Months)	VAS Pain		SSV		ASES		QuickDash	
				Pre	Post	Pre	Post	Pre	Post	Pre	Post
1	F	41	49	7	3	10%	80%	23.3	78.3	81.8	18.2
2	F	30	29	8	0	50%	90%	30	100	90.9	2.3
3	M	42	13	10	1	25%	60%	10	35	88.6	47.7
4	F	38	10	7	0	20%	90%	41.6	90	72.7	17.5
5	F	47	15	8	2	20%	85%	18.3	55	77.3	25
6	M	49	12	8	0	20%	95%	24	90	71	8
7	F	58	6	9	1	10%	80%	20	82	82	23
8	M	20	19	10	1	25%	95%	10	89.9	84.1	6.8
9	F	56	24	9	3	30%	75%	13.4	76.6	93.2	20.5
10	M	41	9	10	6	20%	50%	8.4	40	95.5	68.2

*M* Male, *F* Female, *VAS* Visual analogue scale, *SSV* Subjective shoulder value, *ASES* American Shoulder and Elbow Surgeons Score, *Pre* Preoperative, *Post* Postoperative

**Table 2:** Comparison between preoperative and postoperative variables

	Preoperative values	Postoperative values	Δ CI_Δ 95%_	P
Pain, main ±DS CI95%	8.6 ± 1.1 [7.803–9.397]	1.7 ± 1.8 [0.418–2.982]	−6.9 [−8.157; −5.643]	0.0057
ASES, main ±DS CI95%	19.9 ± 9.8 [12.839–26.961]	73.7 ± 21.4 [58.393–88.967]	53.780 [41.952; 65.608]	< 0.01
Quick Dash, main ±DS CI95%	83.7 ± 8 [78.009–89.411]	23.7 ± 19 [10.096–37.344]	−59.990 [−71.662; −48.318]	< 0.01
SSV, main ±DS CI95%	23 ± 10.8 [15.3–30.7]	80 ± 14.1 [69.9–90.1]	57 [0.452–0.688]	0.00586

## Discussion

The results of our study seem to indicate that endoscopic release of the Pm from the subdeltoid space is a safe and effective procedure with predictable improvements in pain and function in the majority of patients presenting with the diagnosis of isolated pectoralis minor syndrome.

The good results obtained with this technique are in part related to a correct operative indication, which is not always easy because, abnormalities of the pectoralis minor may present with symptoms related to scapulothoracic abnormal motion (STAM), symptomatic compression of the brachial plexus and/or axillary vessels or both. For patients presenting with a compressive brachial plexopathy with or without vascular compression, it is important to identify possible additional sites of compression, including the interscalene triangle and the costoclavicular space. As such, we classify the pectoralis minor syndrome in three grades:

1. **Grade I (muscular Pm syndrome**). Pm abnormalities associated to isolated STAM without neurologic symptoms.
2. **Grade II (neurological Pm syndrome**). Pm abnormalities associated to intermittent or constant compressive brachial plexopathy, with or without STAM or vascular compression.
3. **Grade III (multifocal thoracic outlet syndrome**). Pm abnormalities contribute to compression of the brachial plexus and/or brachial vessels in the setting of double or triple crush at the interscalene triangle or costoclavicular space.

Grade I Pm syndrome is not commonly considered in their differential diagnosis by orthopedic surgeons, but it has been discussed in the past in the physical therapy literature [16], with several stretching protocols reported [15]. Sanchez-Sotelo has compared the Pm as a “hand controlling the scapular position through a joystick, the coracoid process” [17]. Shortening and contracture of the Pm may lead to STAM, anterior scapular tilt, secondary impingement, and when severe it may limit motion.

Provencher et al. reported the outcome of open Pm release specifically for scapular dyskinesis [14]. Their study included 46 shoulders followed for a mean of 25.2 months (range, 24 to 29 months). The majority of the shoulders responded to conservative treatment, and only six shoulders were treated surgically with diagnostic arthroscopy and open pectoralis minor release. These six shoulders experienced improvement in pain (VAS 5.9 preoperatively and 0.9 after surgery), SANE scores (40% preoperatively and 90.4% at most recent follow-up), and ASES scores (improved from 48 to 89 points). The posttreatment outcomes for the operative group could show a significant improvement of the ASES score (from 48 to 89), SANE score (from 40 to 90.4) and VAS (from 5.9 to 0.9). The results of our study are consistent with the outcomes reported by Provencher et al. [14].

The outcome of surgical management for Pm syndrome grades II and III has been classically reported in the vascular surgery literature. In 2011, Sanders reported good to excellent results with isolated open Pm release when treating thoracic outlet syndrome [20]. Later, the same author reported the results of surgical treatment for neurogenic Pm syndrome in children between 11 and 19 years old [18]. In this younger population, 25% of the shoulders were diagnosed as grade II (isolated Pm syndrome) and 75% as grade III [18]. As stated by the authors, the difference between these two degrees far exceeds academic interest, since Pm release is much less invasive that extensile thoracic outlet decompression with first rib resection [18]. A total of 4 studies have reported the outcome of isolated open Pm release for patients with symptoms consistent with thoracic outlet syndrome [2, 18, 21, 23]. Reported good and excellent results in this study have ranged between 50% up to 90%, with high failure rates for shoulders where multifocal compression was not identified or addressed. As such, it is extremely important to carefully evaluate patients with Pm syndrome to separate grade II from grade III shoulders.

Hendrix et al. has described release of the Pm from the glenohumeral joint through the rotator cuff interval [5]. Although such technique is attractive for surgeons with limited experience with endoscopic subdeltoid shoulder surgery, we believe the technique reported in this article is straightforward. Additionally, it provides the potential for a more extensive procedure with formal brachial plexus neurolysis, which requires advanced surgical skills but may provide good results [7, 8]. The relative indications of isolated Pm release versus Pm release combined with brachial plexus neurolysis needs further delineation. However, the results of our study clearly indicate that patients with Grade II Pm syndrome can improve with simple release of the Pm without additional neurolysis. In any event, care must be taken when performing these endoscopic procedures to avoid iatrogenic nerve and especially vascular injuries, which could be devastating.

Our study is not without limitations. We report on a relatively small number of shoulders treated by four different orthopedic surgeons on over a 13-years period, with a variable follow-up time. In addition, the postoperative physiotherapy protocol was not performed in the same center. Moreover, 4 patients present other issues that could influence the symptoms. However, this is the largest study reported to date on arthroscopic release of the Pm for this syndrome, and the technique described was used consistently by the four surgeons who operated on these shoulders.

## Conclusions

Contracture, shortening or hypertrophy of the pectoralis minor may present with a variety of symptoms grouped under the term pectoralis minor syndrome. These symptoms may include abnormal scapulothoracic motion, periscapular pain, pericoracoid pain, intermittent upper limb paresthesia or classic thoracic outlet syndrome. Endoscopic release of the tendon of the pectoralis minor from the coracoid represents a safe and relatively straightforward procedure that improves pain, function and patient reported outcomes in the majority of patients presenting with the diagnosis of isolated pectoralis minor syndrome.

## Supplementary Information

**Additional file 1.** Surgical technique for endoscopic release of the tendon of the pectoralis minor from the coracoid.
